# Invasive songbirds show greater heat, but not cold, tolerance than Mediterranean native counterparts

**DOI:** 10.1242/jeb.252045

**Published:** 2026-06-22

**Authors:** Elena García López de Haro, Erick González-Medina, Julián Cabello-Vergel, Marta Precioso, José A. Masero, Auxiliadora Villegas, Jorge S. Gutiérrez

**Affiliations:** ^1^ Ecology in the Anthropocene, Faculty of Sciences, University of Extremadura, 06006 Badajoz, Spain; ^2^ Conservation Biology Research Group, Faculty of Sciences, University of Extremadura, 06006 Badajoz, Spain; ^3^ Faculty of Biological Sciences, Complutense University of Madrid, 28040 Madrid, Spain

**Keywords:** Cold stress, Heat stress, Invasive species, Mediterranean basin, Passerines, Thermal physiology

## Abstract

Global climate change and biological invasions rank among the leading threats to biodiversity. Rising temperatures can promote invasions by favouring ectothermic species capable of maintaining physiological performance across broader thermal ranges, yet comparative evidence in endotherms remains limited. Here, we examined the heat and cold tolerance limits of three (sub)tropical invasive passerine species [common waxbill (*Estrilda astrild*), red avadavat (*Amandava amandava*) and yellow-crowned bishop (*Euplectes afer*)] and three temperate native passerine species of comparable size and ecology co-occurring in the Mediterranean basin [goldfinch (*Carduelis carduelis*), serin (*Serinus serinus*) and great tit (*Parus major*)]. To assess heat tolerance, we exposed individuals to increasing air temperatures (*T*_a_) during summer and measured their resting metabolic rate, evaporative water loss, the ratio of evaporative heat loss to metabolic heat production, and subcutaneous temperature. During winter, we measured cold tolerance as the minimum tolerated *T*_a_ (in a helium–oxygen atmosphere) and the associated changes in core temperature. As predicted, invasive species exhibited higher heat tolerance limits than native species, associated with a delayed onset of evaporative responses and higher evaporative scopes under extreme heat. Moreover, invasive species made greater use of facultative hyperthermia. However, cold tolerance limits did not differ significantly between invasive and native species. These results indicate that some invasive songbirds possess enhanced heat tolerance while maintaining comparable cold performance, which may allow them to occupy broader thermal environments than their temperate native counterparts. Our findings highlight that physiology plays a substantial role in the success of invasive endotherms.

## Teaser

**Summary:** Examination of co-occurring Mediterranean native and introduced songbird species shows that invasives tolerate higher temperatures than natives, which could explain their success under climate warming.

## INTRODUCTION

Climate change and biological invasions are among the most significant drivers of global biodiversity loss (Dukes and Mooney, 1999; Pyšek et al., 2020). These processes are closely intertwined, because climate change can facilitate the establishment and spread of invasive alien species while amplifying their ecological impacts on native communities (Dukes and Mooney, 1999; Solarz et al., 2023). However, the combined effects of climate change and biological invasions remain poorly understood (Janion-Scheepers et al., 2018; Sala et al., 2000). As global temperatures continue to rise, the ability of species to withstand thermal stress becomes a key determinant of their ecological performance and survival (Conradie et al., 2020; IPCC, 2023; Kearney et al., 2009; Khaliq et al., 2017; Kingsolver et al., 2013; McKechnie and Wolf, 2010), influencing both native and invasive species.

Understanding how invasive species respond physiologically to thermal challenges is fundamental to predicting their range expansion under future climate scenarios (Chown and Gaston, 2008). Invasion success is often associated with a species' ability to tolerate a wide range of thermal conditions, particularly temperature extremes (Zerebecki and Sorte, 2011). Indeed, the ‘greater eurythermality hypothesis’ posits that invasive species generally exhibit broader thermal tolerance ranges than their native counterparts (Kelley, 2014). Empirical evidence supports this hypothesis, showing that invasive species are more likely to maintain physiological function across broader temperature gradients (Janion-Scheepers et al., 2018; Pertierra et al., 2021; but see Barahona-Segovia et al., 2016; Barker et al., 2018; Escribano-Álvarez et al., 2024). This capacity can ultimately determine their ability to establish and persist in novel climatic niches, a recurring feature among successful invaders (Litmer and Murray, 2019; Liu et al., 2022).

Quantifying how invasive species exhibit broader thermal tolerances requires a mechanistic assessment of thermal tolerance, including the upper and lower physiological limits that define a species' thermal niche (Pörtner, 2002). Evidence – largely derived from ectothermic taxa – indicates that invasive species often exhibit thermal tolerance limits exceeding those of native species, especially under heat stress, whereas native species tend to display greater cold tolerance or reveal no significant differences compared with invasive species (Zerebecki and Sorte, 2011; Janion-Scheepers et al., 2018; Pertierra et al., 2021; but see Bujan et al., 2021). Although thermal limits have been extensively documented in endothermic taxa, direct interspecific comparisons in the context of biological invasions remain extremely limited, particularly in birds (but see Sentís et al., 2025 for an intraspecific comparison). This gap hinders our understanding of whether invasive and native endotherms differ in heat and cold tolerance in ways similar to those reported for ectotherms, and how invasive avian species cope with thermal challenges relative to native taxa.

Among endotherms, birds represent one of the most diverse and globally distributed vertebrate groups, occupying a wide range of thermal environments (Jetz et al., 2012). Nevertheless, small passerines can be particularly vulnerable to thermal extremes owing to their high surface-area-to-volume ratio, elevated mass-specific metabolic rates, and limited capacity for heat dissipation (Albright et al., 2017; McKechnie et al., 2021; Payne et al., 2023). Avian thermoregulation relies heavily on evaporative cooling and, consequently, on water availability, which often decreases during periods of extreme heat. This trade-off between heat dissipation and water balance may limit birds' ability to maintain thermal homeostasis, increasing their susceptibility to dehydration and overheating (Dawson, 1982). Given that heatwaves are intensifying in frequency and severity, physiological constraints on passerines may make them increasingly vulnerable under future climate warming scenarios (Cabello-Vergel et al., 2022; IPCC, 2023).

Physiological sensitivity to heat, including traits related to heat tolerance, is increasingly recognized as a robust predictor of avian vulnerability to climate warming (Williams et al., 2008). When air temperature exceeds body temperature, evaporation becomes the only avenue by which birds can dissipate heat (Dawson, 1982; McKechnie and Wolf, 2019). In passerines, this is achieved primarily through panting, a mechanism with high water and energy costs (McNab, 2002; Wolf and Walsberg, 1996) that may ultimately constrain survival. Other evaporative cooling strategies, such as gular fluttering, are generally more energetically efficient (McKechnie et al., 2021). In addition, under hot, humid conditions, the reduction in the water vapour pressure gradient between the animal and the environment can limit evaporative heat dissipation (Gerson et al., 2014) and increase hyperthermia risk. Consequently, the capacity to increase evaporative water loss from basal levels (evaporative scope) is a key determinant of heat tolerance (Czenze et al., 2020) that is physically limited by the interaction between temperature and humidity (Freeman et al., 2024).

Similarly, cold extremes play a critical role in avian persistence, particularly for tropical-origin species encountering thermally variable temperate zones with harsh winters (Keller et al., 2020; Pacioni et al., 2023). In this context, according to the climatic variability hypothesis, organisms inhabiting regions with greater climatic variation evolve broader thermal tolerance ranges (Janzen, 1967). Consistent with this prediction, tropical bird species often cannot tolerate temperatures as low as those tolerated by temperate species, which are adapted to colder environments (Wiersma et al., 2007).

In this study, we compared thermal tolerance limits in six species of native and invasive passerines that coexist in the Mediterranean basin. Based on previous literature and the (sub)tropical origin of the invasive species, we hypothesized that invasive and native species would differ in their thermal capacities. Specifically, we predicted that invasive species would display higher heat tolerance but lower cold tolerance compared with their native counterparts. Such a divergence in thermal physiology may confer an adaptive advantage in increasingly warmer Mediterranean climates.

**Table:** 

**List of symbols and abbreviations**	
CTL	cold tolerance limit
EHL	evaporative heat loss
EWL	evaporative water loss
HTL	heat tolerance limit
*M*_b_	body mass
MHP	metabolic heat production
PIT	passive integrated transponder
RMR	resting metabolic rate
*T*_a_	air temperature
*T*_core_	core temperature
*T*_sub_	subcutaneous temperature
*V*_O_2__	oxygen consumption

## MATERIALS AND METHODS

### Study site and bird captures

The study was conducted in the surroundings of Badajoz (southwestern Spain, 38°56′N, 6°56′W). The region experiences a continental Mediterranean climate, characterized by hot, dry summers and cool winters. During summer, the average daily maximum temperatures reach 36.1±4.0°C (mean±s.d.), with maximum air temperatures peaking up to 45.0°C, whereas in winter, the average daily minimum air temperature is 5.0±3.8°C (mean±s.d.) (Fig. S1) (Spanish State Meteorological Agency, https://opendata.aemet.es).

We evaluated thermal tolerance as follows: (1) heat tolerance limit (HTL), defined as the maximum air temperature (*T*_a_) an organism can tolerate before the onset of severe heat stress indicated by continuous escape behaviour, loss of balance or righting response, rapid declines in oxygen consumption (*V*_O_2__) or evaporative water loss (EWL), and/or a sharp increase in body temperature exceeding 0.1°C min^−1^ near or above 45°C (O'Connor et al., 2021; Whitfield et al., 2015); and (2) cold tolerance limit (CTL), defined as the *T*_a_ at which a bird begins to show hypothermia. Furthermore, we measured several thermoregulatory variables across a thermal gradient, including metabolic rate, EWL, the ratio of evaporative heat loss (EHL) to metabolic heat production (MHP) (EHL/MHP), and body temperature [measured as core temperature (*T*_core_) or subcutaneous temperature (*T*_sub_) depending on the experimental protocol; see below]. Heat tolerance data for native species were obtained from a previous study conducted in the same area (Cabello-Vergel et al., 2022), whereas new data were collected for invasive species following identical methods. Data collection for the heat tolerance of invasive species took place during the summer months (June–September) of 2024, whereas cold tolerance for both invasive and native species was assessed during the winter of 2025 (January–March). We studied six passerine species: three invasive alien species – common waxbill [*Estrilda astrild* (Linnaeus 1758); heat tolerance: *N*=16, cold tolerance: *N*=9], red avadavat [*Amandava amandava* (Linnaeus 1758); heat tolerance: *N*=12, cold tolerance: *N*=9] and yellow-crowned bishop [*Euplectes afer* (Gmelin 1789); heat tolerance: *N*=13, cold tolerance: *N*=7] – and three native species with comparable size and ecological niches – goldfinch [*Carduelis carduelis* (Linnaeus 1758); heat tolerance: *N*=16, cold tolerance: *N*=5], serin [*Serinus serinus* (Linnaeus 1766); heat tolerance: *N*=11, cold tolerance: *N*=3] and great tit (*Parus major* Linnaeus 1758; heat tolerance: *N*=16, cold tolerance: *N*=5). The invasive species originated from sub-Saharan Africa (common waxbill, yellow-crowned bishop) and from the Indian subcontinent and Southeast Asia (red avadavat). The common waxbill, red avadavat and yellow-crowned bishop are non-native passerines well established across the Iberian Peninsula. Their presence is widely associated with accidental escapes from the international pet trade (Carrete et al., 2012; de Lope et al., 1985). These species coexist with native birds (Cardoso and Reino, 2018; Sullivan et al., 2015; Velasco et al., 2017) in habitats such as wetlands, riparian corridors, agricultural mosaics and peri-urban areas (Gregory et al., 2007; Shupova et al., 2024).

We captured invasive birds from each species using mist nets during the summer of 2024 and both invasive and native species during the winter of 2025. To minimize the potential variation in thermal insulation associated with plumage condition, individuals undergoing active moult were excluded from the experiments. Specifically, adults showing active moult of wing or tail feathers, as well as individuals exhibiting heavy body moult, were not included in the measurements, whereas juveniles were only considered when they had completed their first-summer plumage (Klaassen, 1995).

Different individuals were used for the heat tolerance and cold tolerance trials. Individuals were ringed, measured – body mass (*M*_b_), wing, tarsus and bill length – and then transported in cloth bags to the bird facilities of the University of Extremadura, Badajoz, located up to 90 km from the capture sites. Transport time did not exceed 60 min. The birds were housed in large indoor aviaries (320×260×255 cm), with *ad libitum* access to water and food until experimental trials were conducted. All trials were performed within 48 h of capture, allowing birds to recover from transport, handling and temperature-sensitive passive integrated transponder (PIT) tag implantation (see below).

Immediately upon completion of the trials, native birds were released at their original capture sites. However, invasive individuals were euthanized at the end of the experiments using CO_2_ overdose, in accordance with current regional regulations and ethical procedures. All procedures were approved by the Bioethical Committee of the University of Extremadura (196/2023) and carried out under government licenses (CN0019/23/INV and CN0027/24/ACA).

### Gas exchange measures

We used an open-flow respirometry system to measure *V*_O_2__ and EWL. The system consisted of a circuit where a flow of ambient dry air, supplied by a compressor equipped with a desiccating membrane (model R-110300, MESTRA), was divided into two channels: one passing through an empty reference chamber (baseline) and the other through the metabolic chamber containing the bird. The active channel was manually selected using a multiplexer (RM-8, Sable Systems International). Airflow was regulated using mass flow controllers (Flowbar FB-8, Sable Systems International) and adjusted according to species *M*_b_ and the intensity of the thermal challenge. Flow rates were selected to maintain fractional oxygen depletion detectable by the O_2_ analyser while keeping chamber humidity below 1 kPa. This maintained water vapour gradients that did not constrain EWL rates while allowing birds to stay calm (Van Dyk et al., 2019; Whitfield et al., 2015).

After exiting the chambers, the excurrent air stream was subsampled and sequentially passed through a water vapour analyser, a carbon dioxide analyser and an oxygen analyser using a field metabolic system (FMS, Sable Systems International). Prior to metabolic trials, all respirometry equipment was periodically calibrated (zeroed and spanned) following the manufacturer's recommendations (see Supplementary Materials and Methods for further details). An analog interface (Sable Systems International) digitized the voltage outputs from the analysers and the calibrated thermistor probe (TC-100, Sable Systems International), which continuously measured *T*_a_ inside the chamber. Data were recorded every second using ExpeData software (version 1.9.14, Sable Systems International).

### Experimental protocol and setup

All experimental trials were conducted during the birds' active phase, with birds in a postabsorptive state. To ensure postabsorptive conditions, food was removed 2 h prior to each trial (Karasov, 1990). Only one bird was tested per trial.

#### Heat tolerance protocol

We assessed HTLs using the same protocol as Cabello-Vergel et al. (2022). Briefly, individuals were weighed (±0.1 g) before and after each trial and placed into a polypropylene metabolic chamber (effective volume=3.9 l). Each chamber contained a 1 cm layer of mineral oil at the bottom to prevent evaporative water loss from excreta, and a wire mesh platform positioned 5 cm above to allow individuals to perch comfortably.

The baseline and metabolic chambers were placed inside a portable cabinet (Sable Systems International; 540×370×400 mm) equipped with a Peltier device (PELT-5, Sable Systems International) that allowed us to adjust *T*_a_. Thus, individuals were exposed to an increasing gradient of *T*_a_ (30–33°C to 37–40°C), followed by 2°C increments when *T*_a_ exceeded 40°C, continuing until the individual reached its HTL.

Airflow ranged from 1000 to 5000 ml min^−1^, with the baseline flow rate set at 1000 ml min^−1^. Each trial included an initial and final 10-min baseline period. The first measurement period lasted for a minimum of 20 min until stabilization of physiological traces (O_2_, H_2_O). Combined with the initial baseline, this protocol provided a total acclimatization period of at least 30 min. We then followed a stepped protocol: after a 5-min intermediate baseline, we recorded metabolic chamber measurements for a minimum of 10 min (10.17±0.71 min) once the target *T*_a_ was reached, continuing until stable O_2_ and H_2_O traces were obtained. This approach, which involves measurement periods per temperature step within the validated range of <20–30 min, yields results equivalent to longer steady-state exposures (Short et al., 2022). The total duration of a trial did not exceed 3 h per individual. To monitor bird behaviour, we used an infrared video camera. Trials were terminated once the bird reached its HTL. Bird behaviour was scored on a scale from 0 to 5, where 0 indicated complete calm and 5 indicated sustained escape behaviour. Measurements taken during periods of high activity were excluded from data analysis. Upon reaching HTL, the bird was immediately removed from the chamber, and ethanol was applied to its legs while positioned in front of a fan to rapidly lower *T*_sub_ to normothermic values. Birds were then hydrated and returned to the aviaries.

To monitor *T*_sub_ during the trials, we inserted PIT tags (BioTherm13 13×2.12 mm, Biomark, USA) into the interclavicular region of each bird. This technique was employed because *T*_sub_ reliably approximates core temperature at high *T*_a_ (O'Connor et al., 2021; see Supplementary Materials and Methods). PIT tags were implanted immediately after capture, ensuring that all individuals experienced a comparable recovery period prior to the metabolic trials. Furthermore, implantation in this region has been shown to be less invasive for measuring body temperature in small birds than other methods (Oswald et al., 2018). Temperature readings were collected every 5 s using a racket antenna (model F201F-ISO, Biomark) and digitalized by an external reader (IS1001 Multiplexing Transceiver System, Biomark).

#### Cold tolerance protocol

To assess CTLs, we followed the methodology described by Pacioni et al. (2023), with modifications. Cold tolerance was individually measured in a helium–oxygen atmosphere (21% O_2_ and 79% He; helox), which enhances thermal conductance while preventing tissue from freezing (Rosenmann and Morrison, 1974). Prior to the start of each trial, cloacal temperature was measured using a digital thermocouple probe (±0.1°C). Unlike in the metabolic trials described above, body temperature could not be monitored reliably with PIT tags, as readings can be affected by electromagnetic interference from the metal chamber used for this protocol. Cloacal measurements are a suitable method for estimating *T*_core_ at low *T*_a_ in small birds, yielding values comparable to those from other internal sensors (Andreasson et al., 2023). After being weighed, birds were placed into metal metabolic chambers (effective volume=2.1 l) with interiors painted black to maximize radiative heat loss from the bird to the chamber walls and reduce reflection of long-wave radiation during helox trials (Swanson et al., 2014). The temperature inside the metabolic chamber was monitored using a digital thermometer with a type K thermocouple probe (±0.1°C; Wigam TFC-201). The temperature-controlled cabinet was initially set to 10°C, during which an 8-min baseline and a 2-min period of stable metabolic measurements were recorded. Birds therefore experienced a 10-min acclimatization period at 10°C before starting the cooling ramp. Subsequently, *T*_a_ was gradually decreased at a rate of −0.3°C min^−1^, until CTL was reached. CTL was defined as the helox temperature at which *V*_O_2__ reached its maximum and subsequently declined, indicating the metabolic endpoint of the trial and the onset of hypothermia under helox conditions (Swanson et al., 1996). To confirm the onset of hypothermia – defined as a drop in body temperature of ≥5°C below normothermic levels (Ruf and Geiser, 2015) – *T*_core_ was measured immediately after removing the bird from the chamber. Each trial concluded with a 6-min baseline recording, and CTL trials lasted a maximum of 1.5 h.

Helox was pumped through both metabolic and baseline chambers using a flow rate of 500 ml min^−1^ for common waxbill, red avadavat and serin, and 600 ml min^−1^ for great tit, goldfinch and yellow-crowned bishop. Because the density of the helox mixture differs from that of ambient air, flow rates were calibrated using a bubble meter, and corrected values were used for *V*_O_2__ analyses in Expedata (see Supplementary Materials and Methods for details).

### Data analyses

#### Heat tolerance protocol

We corrected O_2_ and H_2_O traces for analyser drift and system lag prior to analyses. Rates of *V*_O_2__ and EWL were then calculated using eqns 10.2 and 10.9 from Lighton (2008). We assumed a respiratory exchange ratio of 0.71, reflecting the postabsorptive metabolic state of the birds (Walsberg and Wolf, 1995) and ensuring direct comparability with the native species dataset reported in Cabello-Vergel et al. (2022). Although CO_2_ was recorded during the trials, we used this fixed RQ value to maintain methodological consistency with that dataset, where metabolic rates were calculated using the same standardized physiological assumption.

Resting metabolic rate (RMR) and EWL were calculated at each target *T*_a_ from the lowest stable 5-min period of *V*_O_2__ and EWL data. These values were converted into watts (W) to estimate MHP, following eqn 9.13 from Lighton (2018), again assuming a respiratory exchange ratio of 0.71. EHL was calculated assuming a latent heat of evaporation of 2.406 J mg^−1^ (Tracy et al., 2010). For each target *T*_a_, *T*_sub_ was averaged over the same 5-min period used for the gas exchange measurements.

For each species, we estimated inflection points for RMR, EWL and *T*_sub_ relative to *T*_a_. These were determined using segmented (broken-stick) linear mixed-effects models implemented with the ‘segmented.lme’ function in the segmented package (Muggeo et al., 2014). Individual identity was included as a random effect to account for repeated measurements across temperature levels, and *M*_b_ was included as a covariate. The slopes above the breakpoints were obtained directly from the segmented models, providing continuous piecewise estimates across the temperature range. To verify that segmented relationships provided a better fit, we compared segmented linear mixed-effects models with standard linear ones using likelihood ratio tests, which supported the use of segmented models (all *P*<0.03; see Table S1). In addition, we calculated the evaporative scope (defined as the ratio between maximum and minimum EWL) as well as the temperature gradient (*T*_sub_−T_a_) at the EWL inflection point. For each parameter, we report the estimate (mean), its standard error (s.e.m.) and the 95% confidence intervals (CIs). Comprehensive details of the segmented mixed-effects models are provided in Table S2.

To account for potential phylogenetic effects on thermal tolerance in interspecific analyses, we constructed a rooted, ultrametric consensus tree based on available avian phylogenies (Jetz et al., 2012; http://birdtree.org) using the ‘phytools’ package in R (Revell, 2012). We then applied generalized linear mixed-effect models fitted via Markov chain Monte Carlo (MCMC) using the ‘MCMCglmm’ package (Hadfield, 2010). Separate models were fitted for each of the following response variables: HTL, maximum EHL/MHP, maximum/minimum RMR, maximum/minimum EWL, temperature gradient at EWL inflection point, with origin (invasive versus native) as the main predictor. *M*_b_ was included as a covariate, and phylogeny as a random effect. For each model, we ran two chains for 5 million iterations, with a burn-in of 100,000 and a thinning interval of 2500. All effective sample sizes exceeded 1000 and autocorrelation values were below 0.1. Convergence was assessed following Gelman and Rubin (1992). We reported significance using posterior distribution probability (proportion of posterior distribution samples that excluded zero; pMCMC) and present estimates and means with 95% CIs.

We used the phylogenetic variance component from each model to estimate heritability (*h*^2^), defined as the proportion of phenotypic variance in thermoregulatory traits attributable to genetic differences (Hadfield, 2010). This measure of phylogenetic signal ranges between 0 (negligible effect of phylogeny) and 1 (strong phylogenetic signal).

#### Cold tolerance protocol

Summit metabolic rates were not included for the cold tolerance trials because the use of helox interfered with the accuracy of the O_2_ and CO_2_ traces under the flow conditions required for these experiments. However, relative changes in *V*_O_2__ remained clearly detectable, allowing us to identify the metabolic endpoint of each trial and determine CTL for all individuals following the established protocol.

We analysed the magnitude of the drop in *T*_core_ (Δ*T*_core_, defined as the difference between the initial *T*_core_ measured before the trial and *T*_core_ after the bird reached a hypothermic state) as a potential thermoregulatory trait indicative of energy-saving capacity (McKechnie and Lovegrove, 2002).

As in the analyses of heat tolerance, we fitted similarly structured generalized linear mixed models using an MCMC framework, with CTL and Δ*T*_core_ as dependent variables and origin (invasive versus native) as the main predictor. *M*_b_ was included as a covariate and phylogeny was incorporated as a random effect. Because residual heteroscedasticity was detected in the CTL model (Goldfeld–Quandt test, *P*=0.06, variance ratio=3.27), heteroscedastic models were preferred over homoscedastic alternatives. Model diagnostics confirmed adequate convergence (Gelman and Rubin, 1992) and the absence of autocorrelation (all values <0.1).

For variables not obtained from fitted models, 95% CIs were estimated via non-parametric bootstrapping (1000 iterations). All statistical analyses were performed in R version 4.4.1 (https://www.r-project.org).

## RESULTS

### Heat tolerance

#### Heat tolerance limits

**Fig. 1.**
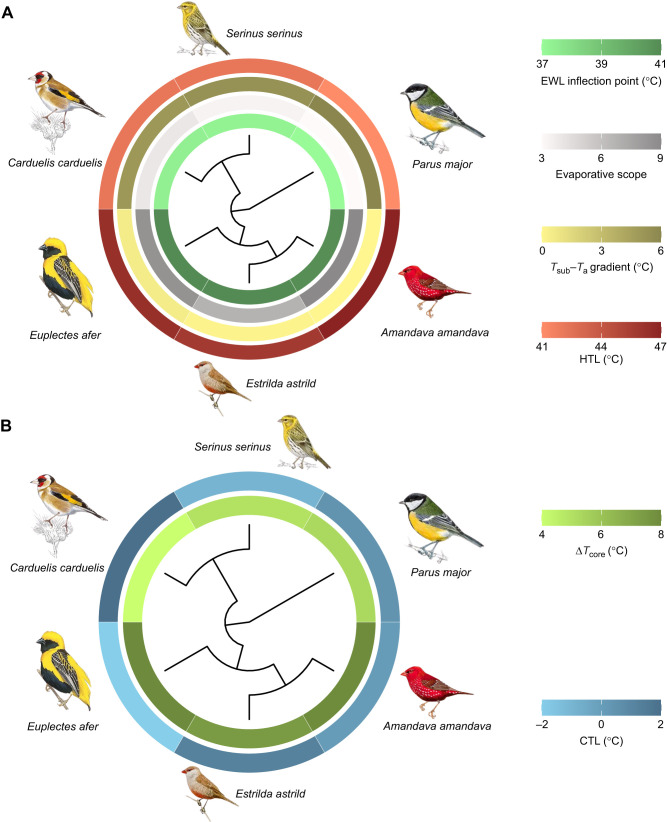
**Consensus phylogenetic tree of native and invasive passerine species included in this study.** (A) Heat tolerance traits measured during summer [inflection point of evaporative water loss, EWL; evaporative scope; temperature gradient between subcutaneous and air temperature (*T*_sub_–*T*_a_) at EWL inflection point; and heat tolerance limit, HTL]. (B) Thermoregulatory traits measured during winter [magnitude of the drop in core temperature (Δ*T*_core_), calculated as the difference between *T*_core_ before the trial and after reaching a hypothermic state; and cold tolerance limit, CTL] (see Tables 1 and 2). Bird illustrations by Juan Varela Simó.

HTLs were significantly higher in invasive species (estimate=6.2°C, 95% CI=2.8, 9.5°C; pMCMC=0.01; Table 1, Fig. 1A). Native species reached HTLs ranging from 36.1°C to 44.1°C, whereas invasive species tolerated substantially higher temperatures ranging from 41.8°C to 50.0°C (Table 2). The difference between normothermic *T*_sub_ and maximum *T*_sub_ at the HTL ranged from 0.6°C to 3.7°C in native species and from 2.5°C to 5.2°C in invasive species (Table 2).

**Table 1.** Results of the model parameters for each thermoregulatory trait analysed in native and invasive songbirds during summer and winter

	Parameter	pMCMC	Estimate	*h* ^2^	Lower 95% CI	Upper 95% CI
Max. EHL/MHP	Origin invasive	0.21	0.28	0.56	−0.26	0.84
	*M*_b_	0.81	−0.01	[0.10, 0.93]	−0.05	0.03
	Intercept	0.02	0.74	–	0.16	1.44
Max./min. RMR	Origin invasive	0.26	0.22	0.18	−0.27	0.72
	*M*_b_	0.10	−0.04	[0.01, 0.71]	−0.10	0.01
	Intercept	<0.001	2.00	–	1.28	2.84
Evaporative scope	Origin invasive	0.02	3.75	0.11	1.39	5.91
	*M*_b_	0.96	0.00	[2e-04, 0.66]	−0.33	0.27
	Intercept	0.04	4.16	–	0.25	8.91
*T*_sub_–*T*_a_ gradient at EWL inflection point (°C)	Origin invasive	<0.001	4.3	0.10	3.8	4.8
	*M*_b_	0.51	0.0	[0.00, 0.57]	−0.0	0.1
	Intercept	0.17	0.5	-	−0.3	1.2
HTL (°C)	Origin invasive	0.01	6.2	0.47	2.8	9.5
	*M*_b_	0.96	−0.0	[0.10, 0.87]	−0.2	0.2
	Intercept	<0.001	41.0	–	37.2	44.9
Δ*T*_core_ (°C)	Origin invasive	0.28	1.0	0.06	−1.1	3.0
	*M*_b_	0.72	0.1	[2e-04, 0.49]	−0.3	0.4
	Intercept	0.02	5.3	–	1.0	9.8
CTL (°C)	Origin invasive	0.11	−3.8	0.00	−10.7	2.0
	*M*_b_	<0.001	−0.8	[0.00, 0.91]	−1.4	−0.4
	Intercept	0.01	12.0	–	4.5	21.8

For each model, we report the posterior distribution probability (pMCMC), the estimate, heritability (*h*^2^), and the lower and upper bounds of the 95% confidence interval (95% CIs for *h*^2^ are provided in brackets below the mean). *M*_b_, body mass; RMR, resting metabolic rate; EWL, evaporative water loss; EHL/MHP, ratio of evaporative heat loss to metabolic heat production; *T*_sub_, subcutaneous temperature; *T*_core_, core temperature; Δ*T*_core_, magnitude of the drop in *T*_core_ (calculated as the difference between *T*_core_ before the trial and after reaching a hypothermic state). For origin level, reference is native.

**Table 2.** Thermoregulatory traits measured in the studied native [serin (*Serinus serinus*), goldfinch (*Carduelis carduelis*) and great tit (*Parus major*)] and invasive species [common waxbill (*Estrilda astrild*), red avadavat (*Amandava amandava*) and yellow-crowned bishop (*Euplectes afer*)] of the Mediterranean songbirds' community

	Native species			Invasive species		
Variable	Serin	Goldfinch	Great tit	Common waxbill	Red avadavat	Yellow-crowned bishop
*M*_b_ (g) summer	10.2±0.2	12.8±0.3	15.5±0.2	7.5±0.1	8.8±0.2	14.6±0.4
RMR						
Min. RMR (W)	0.26±0.02 [0.24, 0.28]	0.29±0.01 [0.27, 0.31]	0.37±0.01 [0.35, 0.40]	0.15±0.01 [0.14, 0.16]	0.17±0.01 [0.16, 0.18]	0.23±0.01 [0.22, 0.25]
Inflection *T*_a_ (°C)	37.1±2.5 [32.3, 41.9]	37.0±1.0 [34.5, 39.4]	35.4±0.8 [33.8, 37.0]	37.6±1.2 [35.3, 39.9]	37.9±0.8 [36.3, 39.5]	38.0±0.6 [36.9, 39.2]
Slope (W °C^−1^)	0.02±0.01 [0.01, 0.03]	0.01±0.00 [0.01, 0.02]	0.04±0.01 [0.02, 0.05]	0.01±0.00 [0.01, 0.02]	0.01±0.00 [0.01, 0.01]	0.02±0.00 [0.01, 0.02]
Max. RMR (W)	0.36±0.02 [0.33, 0.40]	0.36±0.02 [0.33, 0.40]	0.49±0.03 [0.44, 0.54]	0.25±0.02 [0.22, 0.28]	0.28±0.01 [0.26, 0.29]	0.38±0.01 [0.36, 0.40]
Max./min. RMR	1.62±0.09 [1.44, 1.78]	1.33±0.06 [1.21, 1.44]	1.55±0.07 [1.44, 1.68]	1.79±0.17 [1.52, 2.13]	1.70±0.09 [1.55, 1.90]	1.65±0.08 [1.52, 1.79]
EWL						
Min. EWL (mg h^−1^)	62.60±3.27 [59.32, 65.87]	86.15±6.43 [74.48, 98.08]	115.90±8.00 [101.50, 131.90]	47.06±3.87 [39.19, 54.25]	52.98±2.30 [49.03, 57.80]	62.89±3.23 [56.99, 68.99]
Inflection *T*_a_ (°C)	36.4±0.7 [35.0, 37.7]	36.5±0.5 [35.5, 37.5]	35.9±0.6 [34.8, 37.0]	42.2±0.3 [41.6, 42.8]	42.0±0.3 [41.5, 42.6]	42.2±0.3 [41.5, 42.9]
Slope (mg h^−1^°C^−1^)	45.14±3.30 [38.35, 51.92]	45.64±2.15 [41.31, 49.96]	71.94±7.47 [56.86, 87.02]	50.57±4.18 [42.25, 58.88]	57.81±2.49 [52.85, 62.77]	78.09±6.59 [64.94, 91.24]
Max. EWL (mg h^−1^)	379.81±27.13 [333.50, 432.80]	437.36±32.22 [383.80, 503.70]	448.22±26.12 [396.10, 493.10]	294.71±20.69 [256.60, 332.30]	446.61±22.25 [381.90, 508.40]	488.08±25.17 [439.90, 529.00]
Max./min. EWL	4.16±0.46 [3.41, 4.99]	4.65±0.38 [3.97, 5.31]	3.90±0.29 [3.38, 4.46]	6.86±0.91 [5.38, 8.87]	8.63±0.71 [7.35, 9.84]	8.22±0.81 [6.75, 9.63]
EHL/MHP						
Min. EHL/MHP	0.25±0.04 [0.20, 0.34]	0.20±0.01 [0.17, 0.23]	0.20±0.01 [0.18, 0.23]	0.19±0.01 [0.16, 0.21]	0.22±0.02 [0.19, 0.25]	0.19±0.02 [0.15, 0.22]
Max. EHL/MHP	0.68±0.05 [0.61, 0.75]	0.77±0.06 [0.68, 0.88]	0.62±0.04 [0.54, 0.70]	0.82±0.07 [0.71, 0.93]	1.10±0.06 [1.01, 1.21]	1.00±0.06 [0.90, 1.11]
*T*_sub_						
Min. *T*_sub_ (°C)	40.8±0.2 [40.6, 41.0]	41.5±0.2 [41.1, 41.9]	41.6±0.1 [41.4, 41.8]	40.4±0.3 [39.7, 40.9]	40.7±0.2 [40.2, 41.1]	39.9±0.2 [39.5, 40.3]
Inflection *T*_a_ (°C)	36.1±1.7 [32.8, 39.5]	35.3±0.9 [33.6, 37.0]	35.1±1.0 [33.1, 37.0]	35.9±0.9 [34.2, 37.6]	37.5±0.6 [36.3, 38.7]	38.2±0.5 [37.2, 39.3]
Slope (°C °C^−1^)	0.4±0.1 [0.2, 0.5]	0.3±0.0 [0.2, 0.3]	0.3±0.1 [0.2, 0.4]	0.4±0.0 [0.3, 0.4]	0.4±0.0 [0.3, 0.4]	0.5±0.0 [0.4, 0.5]
Max. *T*_sub_ (°C)	43.1±0.2 [42.8, 43.4]	43.1±0.2 [42.7, 43.5]	43.1±0.2 [42.8, 43.4]	44.3±0.2 [43.9, 44.6]	44.5±0.2 [44.1, 44.8]	43.7±0.3 [43.2, 44.2]
Max.–min. *T*_sub_ (°C)	3.2±0.2 [3.0, 3.7]	1.8±0.3 [1.3, 2.4]	1.7±0.2 [1.3, 2.0]	3.8±0.3 [3.3, 4.3]	3.9±0.3 [3.7, 4.9]	4.1±0.3 [3.6, 4.6]
HTL (°C)	41.7±0.3 [41.1, 42.3]	41.7±0.3 [41.0, 42.3]	40.1±0.3 [39.5, 40.5]	46.4±0.3 [45.9, 46.9]	48.2±0.6 [46.9, 49.1]	47.3±0.3 [46.8, 47.8]
*M*_b_ (g) winter	10.8±0.3	12.3±0.4	16.0±0.5	8.3±0.2	9.1±0.2	14.0±0.4
*T*_core_ at the hypothermic endpoint (°C)	34.7±1.0 [33.1, 36.6]	35.1±0.9 [33.7, 36.6]	34.8±0.4 [34.1, 35.4]	33.5±0.6 [32.5, 34.5]	33.7±0.5 [32.6, 34.8]	34.5±0.7 [33.1, 35.9]
CTL (°C)	−1.7±1.1 [−2.9, 0.4]	2.9±1.8 [−0.0, 6.1]	0.4±0.4 [−0.2, 1.3]	1.7±0.4 [1.0, 2.3]	−0.1±0.5 [−0.9, 0.8]	−3.4±0.9 [−5.1, −1.7]
Δ*T*_core_ (°C)	6.0±0.6 [4.9, 6.9]	5.7±1.1 [3.8, 7.5]	6.1±0.5 [5.2, 7.0]	6.8±0.6 [5.7, 7.8]	7.0±0.8 [5.7, 8.7]	7.0±0.8 [5.7, 8.5]

*M*_b_, body mass; RMR, resting metabolic rate; EWL, evaporative water loss; EHL/MHP, ratio of evaporative heat loss to metabolic heat production; *T*_sub_, subcutaneous temperature; *T*_core_, core temperature; Δ*T*_core_, magnitude of the drop in *T*_core_ (calculated as the difference between *T*_core_ before the trial and after reaching a hypothermic state). Data are means±s.e.m. [95% CIs].

#### Subcutaneous temperature during heat exposure

**Fig. 2.**
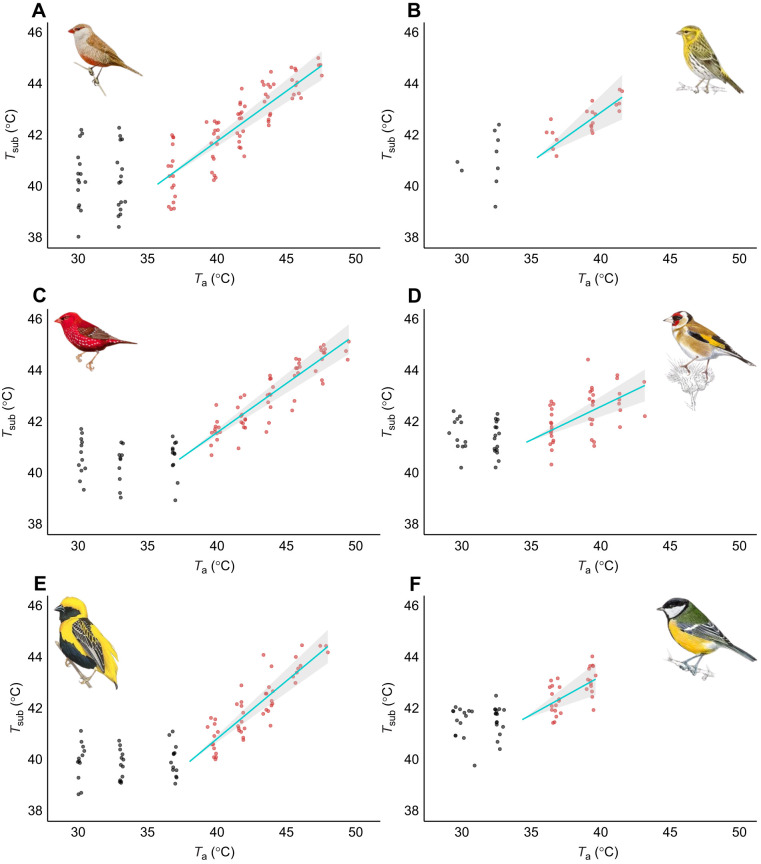
**Subcutaneous temperature (*T*_sub_) as a function of air temperature (*T*_a_) in native and invasive passerine species.** (A) Common waxbill, (B) serin, (C) red avadavat, (D) goldfinch, (E) yellow-crowned bishop and (F) great tit. Linear regressions between *T*_sub_ and *T*_a_ were plotted beginning at the inflection point (see Table 2), with lines showing slopes above the inflection point and shaded grey indicating the 95% confidence interval. Bird illustrations by Juan Varela Simó.

*T*_sub_ inflection points spanned from 35.9°C to 38.2°C in invasive birds and from 35.1°C to 36.1°C in native ones (Table 2, Fig. 2; Fig. S2A). The 95% CI for these inflection points showed overlap between the two groups, indicating that the differences were not significant (95% CI for invasive species: 34.2, 39.3°C; 95% CI for native species: 32.8, 39.5°C; Table 2). The rate of increase in *T*_sub_ in response to *T*_a_ above inflection did not differ between groups, ranging from 0.3°C °C^−1^ to 0.4°C °C^−1^ across native species (95% CI=0.2, 0.5°C °C^−1^) and from 0.4°C °C^−1^ to 0.5°C °C^−1^ in invasive species (95% CI=0.3, 0.5°C °C^−1^; Table 2).

#### Resting metabolic rate

**Fig. 3.**
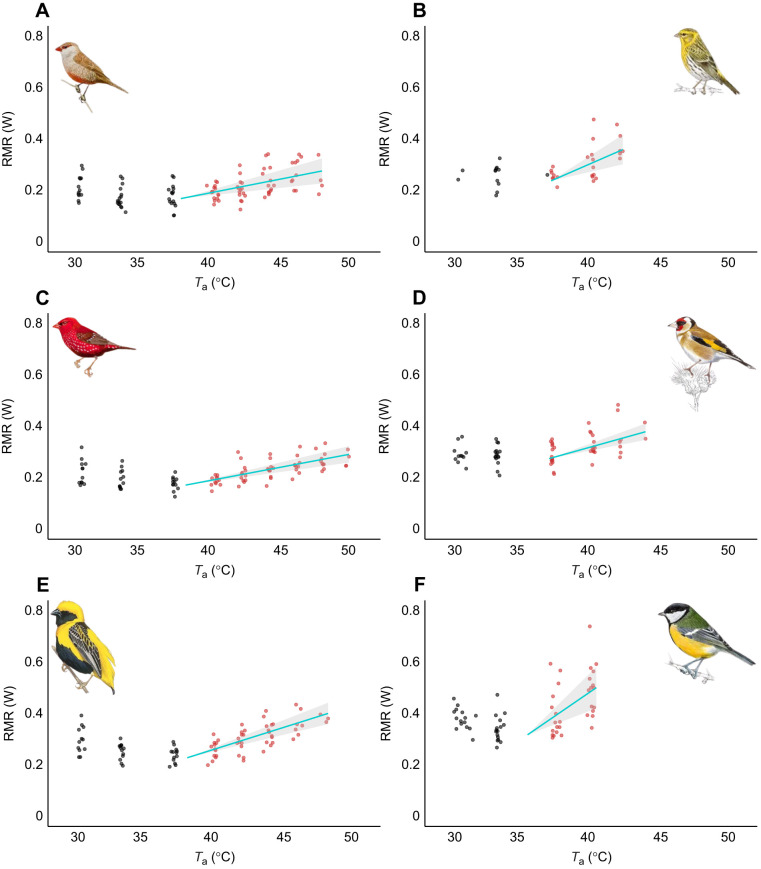
**Resting metabolic rate (RMR) as a function of air temperature (*T*_a_) in native and invasive passerine species.** (A) Common waxbill, (B) serin, (C) red avadavat, (D) goldfinch, (E) yellow-crowned bishop and (F) great tit. Linear regressions between RMR and *T*_a_ were plotted beginning at the RMR inflection point (see Table 2), with lines showing slopes above the inflection point and shaded grey indicating 95% confidence interval. Bird illustrations by Juan Varela Simó.

The RMR inflection point was similar between invasive and native birds, with overlapping 95% CI (invasive species: 35.3, 39.9°C; native species: 32.3, 41.9°C; Table 2; Fig. S2B). Observed values ranged from 37.6°C to 38.0°C in invasive birds and from 35.4°C to 37.1°C in native species (Table 2, Fig. 3).

Above the RMR inflection point, the slope of RMR relative to *T*_a_ did not differ between groups, as indicated by overlapping 95% CI (invasive species: 0.01–0.02 W °C^−1^, 95% CI=0.01, 0.02 W °C^−1^; native species: 0.01–0.04 W °C^−1^, 95% CI=0.01, 0.05 W °C^−1^; Table 2). These results indicate comparable rates of metabolic increase above the RMR inflection point.

#### Evaporative water loss

**Fig. 4.**
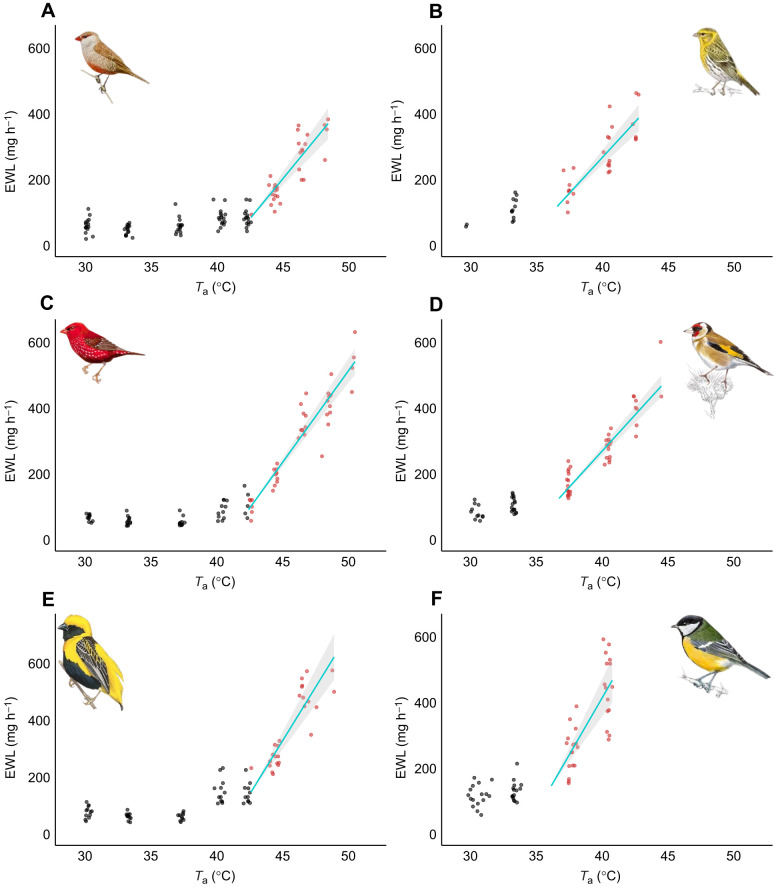
**Evaporative water loss (EWL) as a function of air temperature (*T*_a_) in native and invasive passerine species.** (A) Common waxbill, (B) serin, (C) red avadavat, (D) goldfinch, (E) yellow-crowned bishop and (F) great tit. Linear regressions between EWL and *T*_a_ were plotted beginning at the EWL inflection point (see Table 2), with lines showing slopes above the inflection point and shaded grey indicating 95% confidence interval. Bird illustrations by Juan Varela Simó.

Invasive birds showed higher EWL inflection points than native ones, with non-overlapping 95% CI (invasives=41.51, 42.87°C; natives=34.80, 37.72°C; Table 2, Figs 1A, 4; Fig. S2C). Observed values ranged from 42.03°C to 42.21°C in invasive species and from 35.92°C to 36.49°C in native species (Fig. 4).

Above the inflection point, the slope of EWL relative to *T*_a_ did not differ between groups, as indicated by overlapping 95% CI (slope range for invasives=50.57–78.09 mg h^−1^ °C^−1^, 95% CI=42.25, 91.24 mg h^−1^ °C^−1^; slope range for natives=45.14–71.94 mg h^−1^ °C^−1^, 95% CI=38.35, 87.02 mg h^−1^ °C^−1^; Table 2).

Evaporative scope was significantly higher in invasive species (estimate=3.75, 95% CI=1.39–5.91; pMCMC=0.02; Table 1, Fig. 1A). The observed values ranged from 2.91 to 14.05 in invasive species and from 2.05 to 6.22 in native species (Table 2).

The *T*_sub_–*T*_a_ gradient at the EWL inflection point differed markedly between groups (estimate=4.3°C, 95% CI=3.8, 4.8°C; pMCMC<0.001, Table 1, Fig. 1A). Native species began to increase EWL when *T*_sub_ was still higher than *T*_a_, showing wider *T*_sub_–*T*_a_ gradients, whereas invasive species initiated evaporative cooling at much smaller gradients (*T*_sub_–*T*_a_ gradient=5.0±0.1°C and 0.6±0.1°C, respectively). At their EWL inflection points, invasive species initiated evaporative cooling at *T*_a_ values close to *T*_sub_ (*T*_a_=41.7–42.3°C; *T*_sub_=40.8–43.8°C), whereas native species reached the EWL inflection point at lower *T*_a_ values (*T*_a_=36.0–36.5°C) while maintaining comparatively higher *T*_sub_ values (*T*_sub_=40.2–42.7°C).

#### Maximum ratio of evaporative heat loss to metabolic heat production

Maximum EHL/MHP did not differ significantly between native and invasive species (estimate=0.28, 95% CI=−0.26, 0.84; pMCMC=0.21; Table 1). Native species reached maximum EHL/MHP values ranging from 0.36 to 1.02, whereas invasive species attained higher values ranging from 0.51 to 1.42 (Table 2). Except for two native individuals, only invasive species reached or exceeded an EHL/MHP ratio of 1, indicating that EHL matched or exceeded MHP. The *T*_a_ at which this threshold was reached averaged 47.1±0.3°C in invasive species, whereas in native species this occurred at 40.9±1.7°C.

### Cold tolerance

#### Cold tolerance limits

We found no significant differences in CTL between invasive and native species (estimate=−3.8°C, 95% CI=−10.7, 2.0°C; pMCMC=0.11; Table 1, Fig. 1B). CTL values ranged from −2.9°C to 7.7°C in native species and from −6.8°C to 3.6°C in invasive species (Table 2). *M*_b_ had a significant negative effect on CTL (estimate=−0.8°C g^−1^, 95% CI=−1.4, −0.4°C g^−1^; pMCMC<0.001; Table 1), indicating that heavier individuals reached hypothermic thresholds at lower temperatures.

#### Core temperature during cold exposure

No statistical significance was detected in the Δ*T*_core_ between invasive and native species (estimate=1.0°C, 95% CI=−1.1, 3.0°C; pMCMC=0.28; Table 1, Fig. 1B). *T*_core_ at the end of the cold trials ranged between 30.8°C and 36.9°C in invasive birds and between 33.1°C and 37.0°C in native ones (Table 2). *M*_b_ did not emerge as a significant predictor of the *T*_core_ drop (Table 1).

## DISCUSSION

Our results indicate that the three (sub)tropical invasive species studied here exhibit greater heat tolerance while maintaining cold tolerance levels comparable to those of the temperate native species. This combination results in a broader thermal tolerance range, which may facilitate their establishment and persistence across diverse thermal environments, including conditions outside the climatic envelope of their native distribution (Strubbe et al., 2013, 2023). These findings support the hypothesis that invasion success may be linked to thermal physiology, particularly traits related to water economy that ultimately define the boundaries of a species' fundamental thermal niche (Pacioni et al., 2023; Sentís et al., 2025). The physiological traits quantified here may therefore provide useful parameters for future mechanistic models of invasion risk, as they define the range of tolerable climates and thus the potential for geographic expansion (Sentís et al., 2023; Strubbe et al., 2023). However, these results should be interpreted as patterns observed in the species examined here rather than as a general physiological property of invasive birds.

Invasive species exhibited significantly higher HTLs, reaching values up to 6°C above those of the native species (Table 1, Fig. 1A). This higher heat tolerance appears to be associated with differences in evaporative cooling responses, including a delayed onset of EWL and a greater evaporative scope at high *T*_a_. Delayed increases in EWL may allow these species to minimize energy and water expenditure until reaching higher air temperatures (Cabello-Vergel et al., 2022; Freeman et al., 2024). The HTLs observed in the invasive species are comparable to those reported for similarly sized passerines inhabiting arid environments (e.g. ∼48°C in the scaly-feathered weaver *Sporopipes squamifrons*, ∼10 g; Whitfield et al., 2015) and are higher than the upper limits of heat tolerance documented for passerines from tropical and temperate mesic habitats (40–45.5°C; Pollock et al., 2021). However, these comparisons should be interpreted with caution, as they involve species studied in different geographic regions and ecological contexts.

Invasive birds showed EWL inflection points several degrees higher than those of native species (Table 2, Fig. 1A), indicating a delayed triggering of active evaporative cooling mechanisms. Postponing the onset of evaporative cooling may reduce water and energetic costs at high *T*_a_, a strategy consistent with those observed in warm- or arid-adapted endotherms that prioritize water conservation under acute heat stress (McKechnie et al., 2017, 2021; O'Connor et al., 2017). Although previous work has suggested that birds from humid environments may upregulate evaporative cooling earlier (Freeman et al., 2024), the delayed onset observed here is consistent with the biophysical constraints imposed by high humidity. The invasive species studied here originate from (sub)tropical habitats where high humidity conditions are common. High humidity reduces the water vapour pressure deficit between body surfaces and the surrounding air, thereby limiting the evaporative heat dissipation (Freeman et al., 2022, 2024). Under such conditions, birds may rely more strongly on alternative thermoregulatory strategies such as facultative hyperthermia.

Invasive species also exhibited more pronounced facultative hyperthermia, a controlled increase in body temperature that facilitates passive heat dissipation (Gerson et al., 2019). This pattern was reflected in the *T*_sub_–*T*_a_ gradient at the onset of evaporative cooling, which differed by more than 4°C between groups (Table 1, Fig. 1A), with *T*_a_ approaching *T*_sub_ in invasive species. Facultative hyperthermia allows birds to store heat transiently and delay evaporative cooling under heat stress, a strategy also documented in desert birds as a water-conservation mechanism (Czenze et al., 2020; Smith et al., 2017). In addition to this pattern, invasive species reached a significantly higher evaporative scope (Table 1, Fig. 1A). By conserving water at moderate *T*_a_, invasive species maintain a physiological reserve that enables substantial evaporative water loss as a last resort to dissipate heat during periods of extreme thermal stress. This was accompanied by higher (though not significant; Table 1) maximum EHL/MHP values, allowing complete dissipation of metabolic heat through EWL in invasive species, but generally not in native species (Table 2). The values of these physiological traits overlap with those reported for desert- and arid-zone passerines (Czenze et al., 2020; McKechnie et al., 2017), suggesting that they may contribute to the higher HTLs observed in invasive species.

In addition to their higher heat tolerance, invasive species maintained cold tolerance levels comparable to those of native temperate birds despite their tropical or subtropical origins (Table 1, Fig. 1B). Our CTL results therefore indicate that tropical-origin passerines can persist under cold conditions once established in temperate climates (CTL for tropical birds: 6.70–19.82°C; Noakes and McKechnie, 2020; Van De Ven et al., 2013; Wiersma et al., 2007; CTL for Afrotropical passerines established in temperate regions: −5.10 to −0.50°C; Pacioni et al., 2023). However, the physiological mechanisms underlying this cold tolerance cannot be resolved with our data. Future studies incorporating measurements of resting and maximal thermogenic capacities (RMR and summit metabolic rate) will be necessary to identify the mechanisms supporting cold tolerance in these species (Noakes et al., 2017; Pacioni et al., 2023). The high interspecific variability observed in CTL further highlights the need for broader sampling and comparative studies to better identify the physiological bases of cold endurance and its potential role in invasion success (Khaliq et al., 2017; Swanson, 2010).

The differences observed between native and invasive species may partly reflect the environmental regimes of their regions of origin. The invasive species examined here originate from open, humid reed-dominated habitats characterized by wide diurnal thermal fluctuations and high operative temperatures. Such environments may favour greater thermal tolerance, potentially influencing both heat and cold physiological responses (Pollock et al., 2021; Sentís et al., 2025). In contrast, as the processes of capture and introduction may impose non-negligible selective pressures on behaviour and underlying physiological traits before establishment (Carrete et al., 2012; Chapple et al., 2012), our invasive populations might not be fully representative of their native-range congeners (Sentís et al., 2025). Accordingly, the physiological patterns reported here may partly reflect the climatic conditions under which these species evolved rather than a universal feature of invasive birds. The broader thermal tolerance observed in invasive species likely translates into important ecological consequences. Because heat often acts as a stronger ecological filter than cold in endotherms (Speakman and Król, 2010), the combination of higher heat tolerance and cold tolerance comparable to native species enables these birds to persist across a wider range of thermal environments (Khaliq et al., 2014). Such physiological capacity may facilitate expansion into new areas and increase competitive pressure on native species under changing climatic conditions. Therefore, incorporating these thermoregulatory traits into mechanistic models could improve predictions of invasion risk and future range dynamics (Sentís et al., 2023; Strubbe et al., 2023).

### Conclusions

Our findings indicate that the studied invasive passerines of (sub)tropical origin are more tolerant of extreme heat than their temperate native counterparts, while being similarly tolerant to extreme cold. This higher heat tolerance appears to be facilitated by the delayed onset of EWL and a greater evaporative scope, enabling invasive species to reach substantially higher HTLs. Combined with comparable cold tolerance, this expanded thermal capacity provides these invasive species with a broader functional niche, potentially enhancing year-round survival and facilitating expansion under accelerating climate warming. By linking thermoregulatory performance to invasion success in the species examined here, our findings highlight the potential role of thermal physiology as an important, yet often overlooked, factor influencing biological invasions. Future research incorporating a wider original biogeographic diversity of invasive species will be essential to determine whether these physiological traits are a general feature of successful species or specific to their evolutionary lineages. Understanding these mechanisms is crucial not only for predicting biological invasions, but also for anticipating how reshaped thermal landscapes under climate change will redefine the physiological boundaries, distributional limits and future resilience of endothermic species.

## Supplementary Material

10.1242/jexbio.252045_sup1 Supplementary information
