# Supplementary material for: Invasive songbirds show greater heat, but not cold, tolerance than Mediterranean native counterparts
Source: J Exp Biol. 2026 Jun 22;229(12):jeb252045. doi: 10.1242/jeb.252045 (PMC13354953; doi:10.1242/jeb.252045)
Supplement: Supplementary information [file jexbio-229-252045-s1.pdf]

SUPPLEMENTARY MATERIALS AND METHODS.

1. Respirometry calibration protocols. Field Metabolic System (FMS).

Calibration of the Field Metabolic System (FMS, Sable Systems International) was performed in accordance with the manufacturer's protocols (Sable Systems International, 2018).

1.2. System Zeroing (water vapour and carbon dioxide analysers).

We performed a system-wide zeroing procedure every two weeks. We flowed pure nitrogen gas (N_2) through the FMS unit at a constant flow rate (~1,000 ml min^{-1}) until water vapour pressure (WVP, kPa) and carbon dioxide (CO_2, %) readings reached a minimum value close to or below zero. Once this occurs, we maintained N_2 flow by 40 min and then these minimum values were digitally set as the zero point for each sensor.

1.3. System spanning (water vapour, carbon dioxide and oxygen analysers).

1.3.1. Water Vapour Span.

The water vapour analyser was calibrated every two weeks by establishing the span using the 'oxygen dilution method', which accounts for fluctuations in barometric pressure (BP). First, air was passed through a Drierite (set before the analysers) column to remove all moisture until oxygen (O_2) and barometric pressure (BP) readings were stable. After recording these dry air values, the Drierite column was removed to allow humid air into the system, and we recorded the new stable O_2 and BP readings under ambient conditions (wet air). We compared the O_2 readings of dry air against wet air to calculate the WVP, as shown in Eqn S1:

$$\mathrm{WVP} = \mathrm{BP} \times \frac{\mathrm{dry\ O_2} - \mathrm{wet\ O_2}}{\mathrm{dry\ O_2}} \qquad \text{Eqn S1}$$

where BP is the average barometric pressure recorded during the dry and wet measurement phases. The resulting value was used to set the span of the humidity sensor.

Journal of Experimental Biology • Supplementary information

1.3.2. Carbon dioxide Span.

The CO_2 sensor was spanned every two weeks using a calibration gas with a known concentration of 3,000 ppm. We flowed this gas through the FMS unit for 5 min until the reading stabilized. Once stabilized, this value was digitally set as the span point for the CO_2 sensor.

1.3.3. Oxygen Analyser Span.

Following the zeroing procedure, the O_2 sensor was spanned using the 'ambient air' method. We drew outside ambient air through the analyser, allowing the flow to continue until the O_2 reading stabilized. We then set the O_2 span to 20.95%, which represents the standard concentration of O_2 in dry atmospheric air. To account for potential sensor drift, this span adjustment was verified daily prior to the commencement of metabolic trials.

The respirometry setup and calibration protocols for the native species are described in detail by Cabello-Vergel et al. (2024).

2. Flow rate calibration for helox

Due to the density difference between helox (79% helium, 21% O_2) and ambient air, the flow rates displayed by mass flow controllers calibrated for air do not correspond to the actual flow rates passing through the metabolic system. To correct this discrepancy, helox flow rates were calibrated using a bubble-meter prior to O_2 consumption analyses in Expedata.

Calibration was performed by directing the helox flow through a graduated column and recording the time required for a soap bubble to travel between two known volume marks. The actual flow rate (ml min^{-1}) was calculated as shown in Eqn S2:

$$\text{Flow rate} = \frac{\text{Volume between marks (ml)}}{\text{Time (min)}} \qquad \text{Eqn S2}$$

The resulting correction factors were then applied to the flow rates used in subsequent O_2 consumption calculations.

Journal of Experimental Biology • Supplementary information

Climatological data of the study area

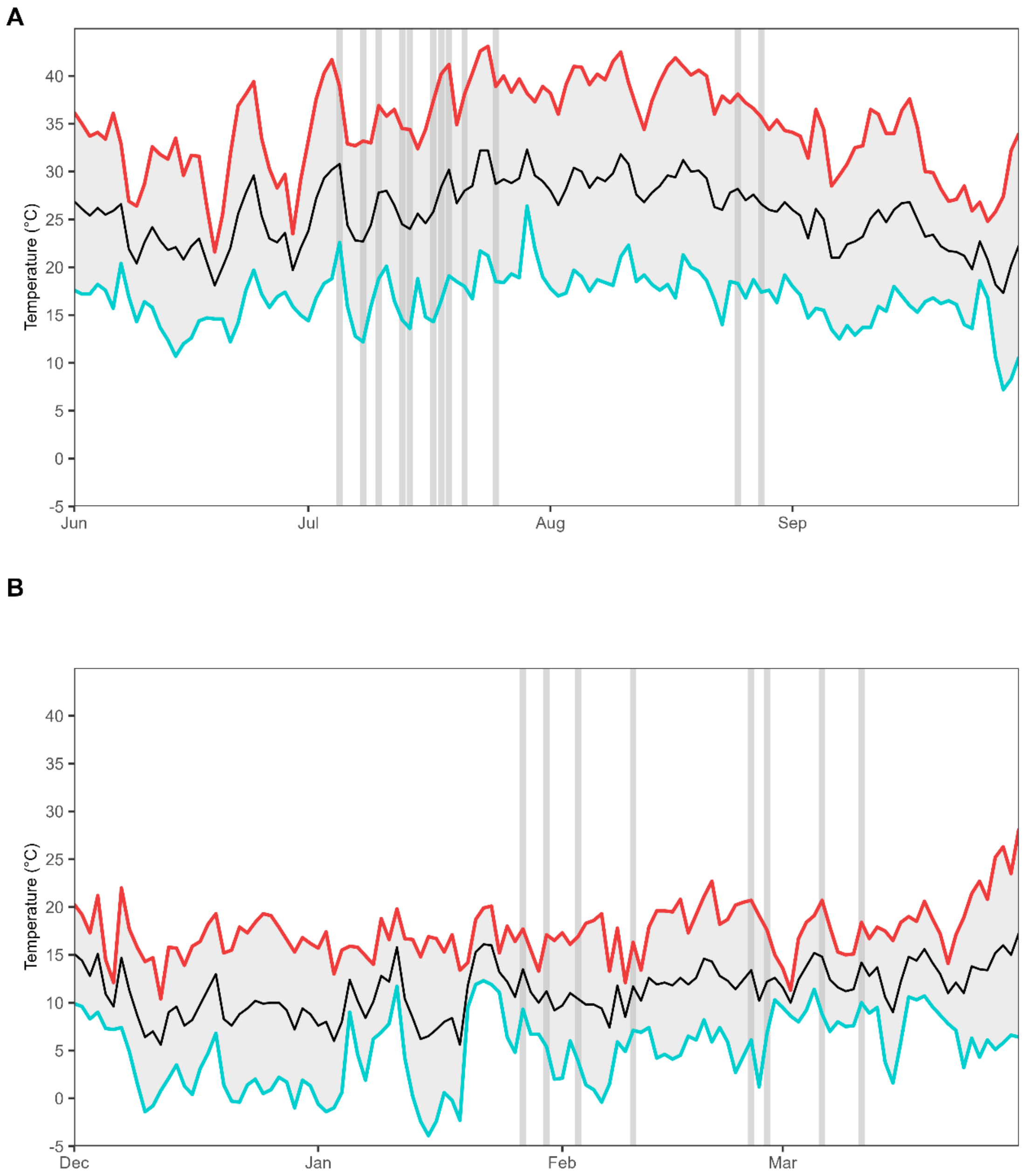

Fig. S1. Maximum (red), minimum (blue), and average (black) daily air temperature variation and capture events during the study periods. (A) Temperatures recorded during the summer of 2024. (B) Temperatures recorded during the winter of 2024–2025. In both panels, vertical grey bars indicate the days on which bird capture events were conducted. Temperature data were obtained from the Spanish State Meteorological Agency (https://opendata.aemet.es).

Journal of Experimental Biology • Supplementary information

Integrative overview of thermal responses to heat in invasive and native species

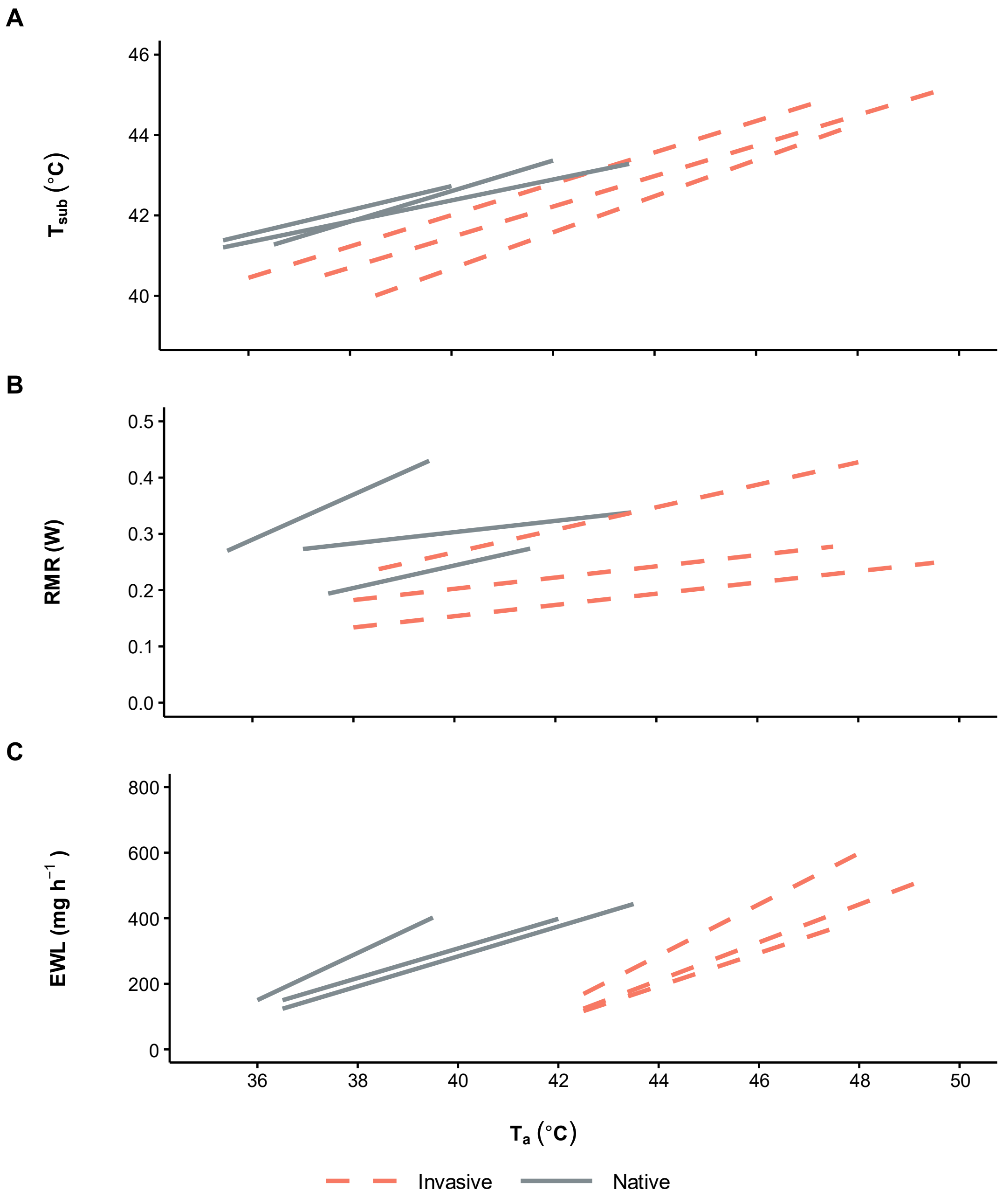

Fig. S2. Thermal response curves of invasive and native passerine species across the experimental T_a gradient. Curves represent changes in (A) subcutaneous temperature (T_{sub}), (B) resting metabolic rate (RMR), and (C) evaporative water loss (EWL) as a function of air temperature (T_a). Invasive species are shown with dashed lines (orange) and native species with solid lines (dark grey). This figure provides an integrative comparison of thermal response patterns under heat stress among both groups.

Journal of Experimental Biology • Supplementary information

Table S1. Likelihood ratio test statistics comparing segmented vs. standard linear mixed-effects models for thermoregulatory traits in native species: Serin (*Serinus serinus*), Goldfinch (*Carduelis carduelis*), and Great tit (*Parus major*); and invasive species: Common waxbill (*Estrilda astrild*), Red avadavat (*Amandava amandava*), and Yellow-crowned bishop (*Euplectes afer*). Chi-squared (χ^2; df = 2), and p-values are reported for each model comparison.

Variable	Species	χ^2	p-value
**RMR (W)**	**Invasive species**		
	Red avadavat	40.03	< 0.001
	Common waxbill	23.94	< 0.001
	Yellow-crowned bishop	53.83	< 0.001
	**Native species**		
	Goldfinch	22.30	< 0.001
	Serin	7.76	0.02
	Great tit	17.19	< 0.001
**EWL (mg h^{-1})**	**Invasive species**		
	Red avadavat	161.93	< 0.001
	Common waxbill	141.27	< 0.001
	Yellow-crowned bishop	109.41	< 0.001
	**Native species**		
	Goldfinch	70.59	< 0.001
	Serin	27.84	< 0.001
	Great tit	32.57	< 0.001
**T_{sub} (ºC)**	**Invasive species**		
	Red avadavat	62.81	< 0.001
	Common waxbill	48.05	< 0.001

Yellow-crowned bishop	64.13	< 0.001
**Native species**		
Goldfinch	19.44	< 0.001
Serin	7.07	0.03
Great tit	13.11	0.01

RMR, resting metabolic rate; EWL, evaporative water loss; T_{sub}, subcutaneous temperature.

Table S2. Results of segmented linear mixed-effects models for thermoregulatory traits in native species: Serin (*Serinus serinus*), Goldfinch (*Carduelis carduelis*), and Great tit (*Parus major);* and invasive species: Common waxbill (*Estrilda astrild*), Red avadavat (*Amandava amandava*), and Yellow-crowned bishop (*Euplectes afer*) from the Mediterranean songbird community. Estimates, standard errors (SE), 95% confidence intervals (CIs), and p-values are reported for the intercept, body mass (M_b, g), breakpoint, and slope.

Variable	Species	Parameter	Intercept	M_b	Breakpoint	Slope
**RMR (W)**	**Invasive species**					
		Estimate	0.37	0.00	37.92	0.01
		SE	0.11	0.01	0.82	0.00
	Red avadavat	l- 95% CI	0.17	-0.01	36.32	0.01
		u-95% CI	0.58	0.02	39.52	0.01
		p-value	0.001	0.65	-	< 0.001
		Estimate	0.20	0.02	37.50	0.01
		SE	0.11	0.01	1.12	0.00
	Common waxbill	l- 95% CI	-0.02	0.00	35.31	0.01
		u-95% CI	0.42	0.04	39.69	0.02
		p-value	0.08	0.04	-	< 0.001

	Yellow-crowned bishop	Estimate	0.53	0.00	38.04	0.02
		SE	0.11	0.01	0.60	0.00
		l- 95% CI	0.33	-0.01	36.85	0.01
		u-95% CI	0.75	0.01	39.22	0.02
		p-value	< 0.001	0.98	-	< 0.001
	**Native species**					
	Goldfinch	Estimate	0.14	0.02	36.95	0.01
		SE	0.16	0.01	1.02	0.00
		l- 95% CI	-0.20	0.01	34.49	0.01
		u-95% CI	0.41	0.03	39.40	0.02
		p-value	0.37	0.005	-	< 0.001
	Serin	Estimate	0.14	0.02	37.08	0.02
		SE	0.37	0.02	1.80	0.01
		l- 95% CI	-0.59	-0.02	33.55	0.01
		u-95% CI	0.86	0.06	40.62	0.04
		p-value	0.72	0.36	-	< 0.001
	Great tit	Estimate	0.53	0.01	35.38	0.04
		SE	0.34	0.01	0.83	0.01
		l- 95% CI	-0.16	-0.01	33.75	0.02
		u-95% CI	1.19	0.04	37.00	0.05
		p-value	0.13	0.34	-	< 0.001
**EWL (mg h^{-1})**	**Invasive species**					
	Red avadavat	Estimate	-148.98	12.28	42.03	57.81
		SE	75.66	6.55	0.26	2.49
		l- 95% CI	-294.23	-0.62	41.51	52.85
		u-95% CI	2.36	25.06	42.55	62.77
		p-value	0.0526	0.09	-	< 0.001
	Common waxbill	Estimate	-193.67	20.89	42.22	50.57

Journal of Experimental Biology • Supplementary information

		SE	52.46	5.76	0.31	4.18
		l- 95% CI	-294.70	9.80	41.61	42.25
		u-95% CI	-89.06	32.36	42.82	58.88
		p-value	0.00	0.00	-	< 0.001
	Yellow-crowned bishop	Estimate	-258.48	5.11	42.21	78.09
		SE	92.76	5.37	0.34	6.59
		l- 95% CI	-436.90	-5.29	41.54	64.94
		u-95% CI	-73.30	15.77	42.87	91.24
		p-value	0.0069	0.36	-	< 0.001
	**Native species**					
	Goldfinch	Estimate	-254.82	12.46	36.49	45.64
		SE	144.30	4.75	0.51	2.15
		l- 95% CI	-554.71	3.16	35.49	41.31
		u-95% CI	10.91	21.78	37.49	49.96
		p-value	0.08	0.02	-	< 0.001
	Serin	Estimate	-321.32	37.11	36.36	45.14
		SE	327.68	25.40	0.70	3.30
		l- 95% CI	-978.35	-12.92	34.99	38.35
		u-95% CI	306.12	86.65	37.72	51.92
		p-value	0.34	0.18	-	< 0.001
	Great tit	Estimate	70.27	-8.35	35.92	71.94
		SE	286.60	10.47	0.57	7.47
		l- 95% CI	-487.24	-28.91	34.80	56.86
		u-95% CI	636.22	12.13	37.04	87.02
		p-value	0.81	0.44	-	< 0.001
**T_{sub} (ºC)**	**Invasive species**					
	Red avadavat	Estimate	40.9	-0.0	37.5	0.4
		SE	2.1	0.2	0.6	0.0

	l- 95% CI	36.9	-0.4	36.3	0.3
	u-95% CI	45.2	0.4	38.7	0.4
	p-value	< 0.001	0.90	-	< 0.001
Common waxbill	Estimate	39.0	0.4	35.9	0.4
	SE	3.7	0.4	0.9	0.0
	l- 95% CI	31.8	-0.3	34.2	0.3
	u-95% CI	46.3	1.1	37.6	0.4
	p-value	< 0.001	0.26	-	< 0.001
Yellow-crowned bishop	Estimate	40.2	-0.0	38.2	0.5
	SE	1.7	0.1	0.5	0.0
	l- 95% CI	36.8	-0.2	37.2	0.4
	u-95% CI	43.5	0.2	39.3	0.5
	p-value	< 0.001	0.79	-	< 0.001
**Native species**					
Goldfinch	Estimate	40.7	0.2	35.3	0.3
	SE	2.5	0.1	0.9	0.0
	l- 95% CI	35.4	-0.0	33.6	0.2
	u-95% CI	45.3	0.4	37.0	0.3
	p-value	< 0.001	0.08	-	< 0.001
Serin	Estimate	40.8	-0.0	36.1	0.4
	SE	6.8	0.4	1.7	0.1
	l- 95% CI	26.6	-0.9	32.8	0.2
	u-95% CI	53.3	0.8	39.5	0.5
	p-value	< 0.001	0.96	-	< 0.001
Great tit	Estimate	40.5	0.0	35.2	0.3
	SE	2.5	0.1	1.0	0.1
	l- 95% CI	35.4	-0.2	33.2	0.2
	u-95% CI	45.2	0.3	37.1	0.4
	p-value	< 0.001	0.73	-	< 0.001

RMR, resting metabolic rate; EWL, evaporative water loss; T_{sub}, subcutaneous temperature.

Journal of Experimental Biology • Supplementary information

SUPPLEMENTARY REFERENCES

Cabello-Vergel, J., Gutiérrez, J. S., González-Medina, E., Sánchez-Guzmán, J. M., Masero, J. A. and Villegas, A. (2024). Seasonal and between-population variation in heat tolerance and cooling efficiency in a Mediterranean songbird. *J. Therm. Biol.* **125**, 103977.

Sable Systems International (2018). *Instruction Manual FMS Field Metabolic System Version 3.0.*
